# Plasma Non-Esterified Fatty Acid Levels Throughout Childhood and Its Relationship with Leptin Levels in Children

**DOI:** 10.3390/jcm13237286

**Published:** 2024-11-30

**Authors:** Olga Pomares, Claudia Vales-Villamarín, Iris Pérez-Nadador, Francisco J. Mejorado-Molano, Leandro Soriano-Guillén, Carmen Garcés

**Affiliations:** 1 Lipid Research Laboratory, IIS-Fundación Jiménez Díaz, UAM, 28040 Madrid, Spain; olga.pomares@quironsalud.es (O.P.); claudia.vales217@gmail.com (C.V.-V.);; 2 Department of Pediatrics, IIS-Fundación Jiménez Díaz, UAM, 28040 Madrid, Spain; fmejorado@quironsalud.es (F.J.M.-M.); lsoriano@fjd.es (L.S.-G.)

**Keywords:** free fatty acids, insulin, obesity, paediatric population

## Abstract

**Background/Objective:** The relationship of non-esterified fatty acid (NEFA) levels with obesity and obesity-related alterations shows age-dependent variability in children. Leptin, with an important role in energy homeostasis and lipid metabolism, may be related to NEFA levels throughout the first decades of life. This cross-sectional study aims to analyse plasma NEFA levels in children of different ages and evaluate the relationship of leptin with NEFA levels depending on age. **Methods**: The study sample included 818 prepubertal children (age 6–8 years) and 762 adolescents (age 13–16 years). NEFA levels were measured using the Wako NEFA-C kit. Insulin and leptin levels were determined by IRMA and ELISA, respectively, using commercial kits. **Results:** The results of the study were found to show that NEFA levels were significantly higher (*p* < 0.001) in prepubertal children than in children aged 13 to 16 years (0.68 ± 0.3 mmol/L vs. 0.42 ± 0.2 mmol/L, respectively, in males; 0.71 ± 0.3 mmol/L vs. 0.44 ± 0.2 mmol/L, respectively, in females), showing a progressive decrease according to years of life in this cohort of adolescent in both sexes. Leptin and insulin correlated negatively with NEFA levels in younger children but not in older participants. The negative association between NEFA levels and leptin occurring in prepubertal children remained significant when adjusting for insulin. **Conclusions:** Besides reporting that NEFA levels decrease between the prepubertal age and adolescence, our findings indicate that, in children aged 6–8 years, leptin is associated with NEFA levels, independently of insulin. However, this relationship is not present in older children. Further studies analysing these associations according to pubertal status would be useful to deepen our understand of these findings.

## 1. Introduction

Research has suggested that non-esterified fatty acids (NEFAs) are the link between obesity and insulin resistance [1]. Increased NEFA levels have been described in adults with obesity [1,2] and have been established as a risk factor for metabolic disease [3]. In children, on the other hand, the association of obesity with increased NEFA levels remains controversial [4,5,6,7,8]. Some studies [4,5,7] have shown significantly lower NEFA levels in children with obesity when compared with normal-weight controls. Conversely, other studies [6] have reported elevated circulating NEFA levels in children with obesity when compared with those of a normal-weight; however, this significant increase was observed exclusively in boys, not girls. We have previously reported significantly lower levels of NEFA in prepubertal girls with obesity than in those without obesity [9], although no differences were found in children with and without obesity between the ages of 12 and 16 years [10].

Leptin plays a prominent role in energy homeostasis and obesity [11] but is also a key factor in adipocyte metabolism, affecting lipogenesis, lipolysis, and fatty acid oxidation [12,13]. Its influence on a variety of physiological processes makes it a molecule of potential interest for clinical and therapeutic applications, particularly in the treatment of obesity and metabolic disorders, such as type 2 diabetes, as well as neurodegenerative diseases and cancer [14].Therefore, the influence of leptin was explored on the association between obesity and NEFA levels in our cohorts of children, finding an influence of plasma leptin on the association between NEFA and obesity in 6-to-8-year-old children but no such influence in adolescents [15], indicating the age-dependent role of leptin.

Leptin is involved in adipocyte metabolism both directly and indirectly [12,16,17]. The direct action exerted on adipocytes occurs through interaction with the leptin receptor, down-regulating lipogenesis and up-regulating lipolysis and fatty acid oxidation [18], while indirect action results from activating sympathetic nerve fibres [16,19]. Though the main regulatory effects of leptin on energy metabolism are exerted by the activation of signalling pathways in the central nervous system, the autocrine effects of leptin, with a crosstalk with insulin, have been established [20]. We hypothesized that any of these effects of leptin may be predominant depending on age.

The available data on NEFA concentrations in the paediatric population is limited. Furthermore, there is a notable lack of studies that analyse NEFA levels across different age groups. As NEFAs play a crucial role in energy metabolism, studying NEFA levels by age will help to elucidate the way in which metabolic processes evolve from infancy to adulthood. Analysing how NEFA levels change with age will also allow an understanding of how NEFA levels are regulated early in life and may offer insights into the origins of obesity and related diseases, potentially leading to early intervention strategies.

Therefore, the aim of our study is to analyse NEFA levels in two population-based cohorts of children of different ages and to evaluate the potential relationship between plasma NEFA and leptin levels in the first decades of life.

## 2. Materials and Methods

### 2.1. Subjects

**Figure 1:**
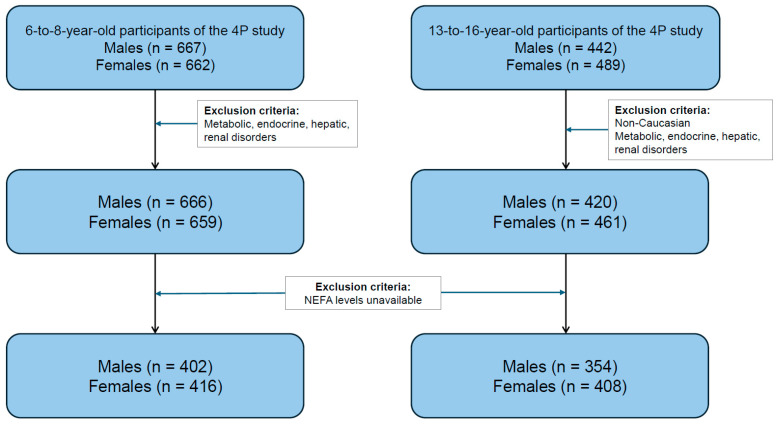
Flowchart diagram detailing subject selection.

This analysis is a sub-study within the comprehensive Four Provinces Study (4P), a cross-sectional examination of cardiovascular risk factors in children [21]. Children were selected through a random cluster sampling of schools, with stratification based on gender and type of school (i.e., distinguishing between public and private institutions) to obtain a representative sample of children from both age groups. Non-Caucasian children were excluded. Parents or legal guardians were asked to report in writing whether their children had any metabolic, endocrine, hepatic, or renal disorders. Children identified with any of these conditions were excluded from the study, as these conditions may confound the interpretation of the study variables. The participants selected for this sub-analysis were those children for whom data on NEFA levels were available (see Figure 1).

Approval for the study protocol was obtained from the Clinical Research Ethics Committee of the Instituto de Investigación Sanitaria Fundación Jiménez Díaz (IIS-FJD) (Approval reference: PIC105-2023 FJD, 15 September 2023). This investigation adhered meticulously to the ethical principles of the Declaration of Helsinki and its subsequent revisions, as well as the prevailing legislation governing clinical research involving human participants in Spain. Written informed consent was obtained from parents or legal guards for their children’s participation in the study. Additionally, assent was obtained from all children over the age of 8.

### 2.2. Data Collection

The same team, which included one physician and several nurses, was responsible for conducting blood extractions and taking physical measurements, such as weight and height.

#### 2.2.1. Anthropometric Measurements

The participating children were measured for weight and height while barefoot and wearing light clothing. Both weight and height were recorded to the nearest 0.1 unit (kg or cm, respectively) using a standardized electronic digital scale and a portable stadiometer, respectively. These measures were used to determine BMI, calculated as weight in kilograms divided by height in meters squared (kg/m^2^).

#### 2.2.2. Biochemical Data

Blood samples were collected by venipuncture in the early morning after a 12 h fasting period and stored in vacutainer tubes. Subsequently, the samples were centrifuged at 1500× *g* at a temperature of 4 °C for 25 min, which separated serum and plasma into aliquots. These aliquots were promptly stored at −70 °C to ensure preservation for subsequent analysis.

NEFAs were measured using the Wako NEFA-C kit (Wako Industries, Osaka, Japan). Insulin levels were determined with a commercial kit (BI-Insulin IRMA, Bio-Rad, Marnes la Coquette, France). Leptin levels were measured by ELISA using a commercial kit (Leptin EIA-2395, DRG, Marburg, Germany).

### 2.3. Statistical Analysis

Statistical analyses were performed using the SPSS software package, version 25.0 (IBM, New York, NY, USA) and the GraphPad Prism version 8 statistical software (San Diego, CA, USA). The Kolmogorov–Smirnov test was used to assess whether the variables under study were normally distributed. We used the Mann–Whitney U test to compare levels of non-normally distributed variables (insulin, NEFA, and leptin) between the prepubertal and adolescent groups, as well as between sexes. Age-based comparisons of the variables under study were conducted using ANOVA, followed by the Games–Howell post-hoc test. Spearman correlation was carried out to evaluate the association between the study variables in both cohorts of children. Partial correlation analyses were conducted to determine the relationship between NEFA and leptin levels after adjusting for insulin levels. Additionally, univariate analyses of variance were performed to further analyse this relationship between leptin (categorized in tertiles) and NEFA levels after adjusting for BMI and insulin levels.

## 3. Results

Two Spanish Caucasian population-based samples were studied: one comprising 818 children (402 males and 416 females) aged 6–8 years and another consisting of 762 adolescents (354 males and 408 females) aged 13–16 years. Characteristics of the population under study by age and sex are described in Table 1. The mean age was 7.1 ± 0.6 years in the group of prepubertal children and 14.5 ± 0.9 years in the adolescents’ group, without significant differences between sexes in any group. No significant differences were found when comparing insulin and NEFA levels between sexes in the adolescent group, while slightly higher levels were observed in prepubertal females as compared with males of the same age group. Leptin levels were significantly higher (*p* < 0.001) in females than in males in both cohorts, which could be explained by a combination of hormonal and metabolic factors.

**Figure 2:**
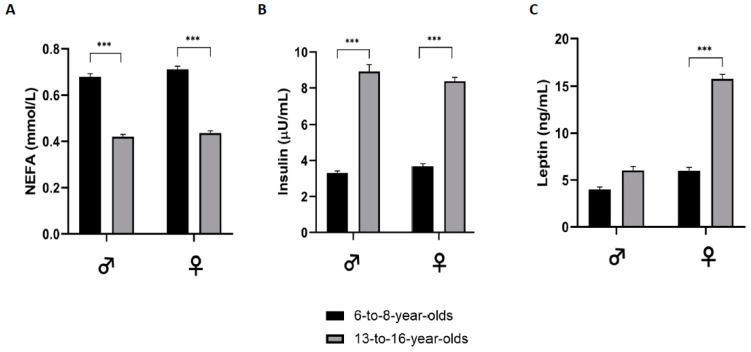
NEFA(**A**), insulin (**B**), and leptin (**C**) levels (means and std. error) by group of age. NEFA—non-esterified fatty acids. *p*-value: *** *p*< 0.001.

As shown in Figure 2, NEFA levels were significantly higher (*p* < 0.001) in prepubertal children when compared with adolescents in both sexes, while insulin levels were significantly lower in prepubertal children (*p* < 0.001) than in the adolescent group. Leptin levels were significantly higher in adolescent females than in prepubertal females (*p* < 0.001), while no significant differences between age groups were found in male participants.

**Figure 3:**
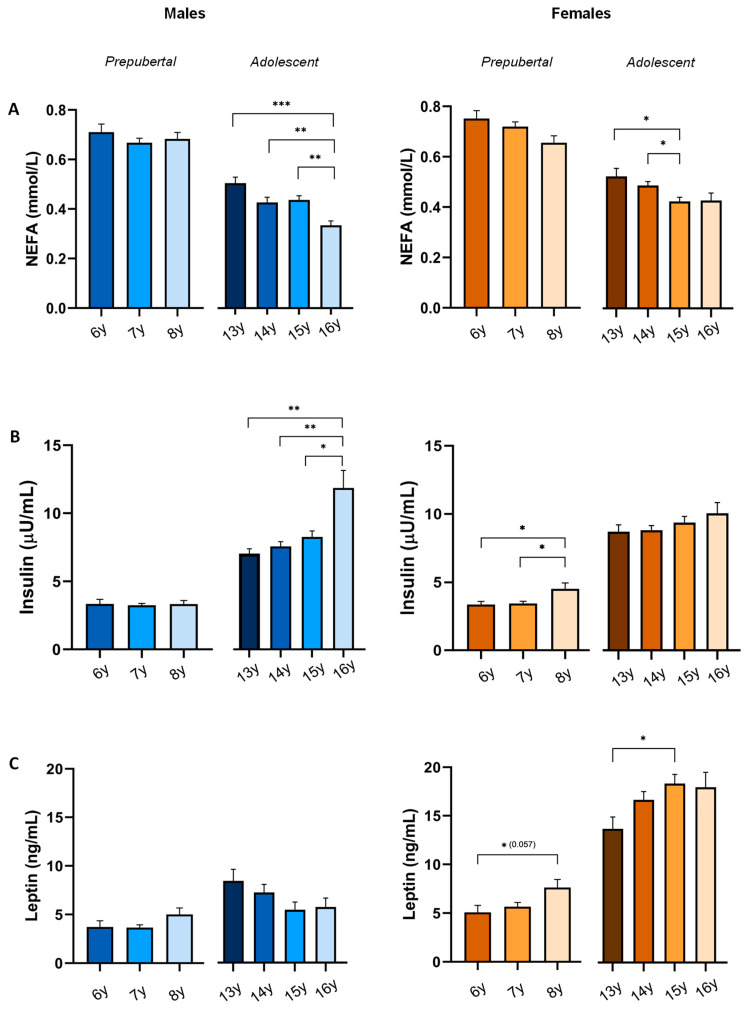
NEFA (**A**), insulin (**B**), and leptin (**C**) levels by age and sex. *p*-value: * *p* < 0.05; ** *p* < 0.01; *** *p*< 0.001.

To further analyse their evolution by age, the variables under study were compared according to age in years within both cohorts (Figure 3). This analysis revealed no significant differences by age in NEFA, insulin or leptin levels among prepubertal males. A progressive decrease in NEFA levels was observed in prepubertal females, although without reaching significant association. In prepubertal females, significantly higher levels of insulin and leptin were observed in girls aged 8 years as compared with 6- or 7-year-olds. On the other hand, in adolescent males, NEFA levels showed a progressive decrease, being significantly lower in 16-year-olds as compared with males of 13, 14 and 15 years. Insulin levels were higher in 16-year-olds compared with males of 13, 14 and 15 years. No significant age-based differences were found in leptin levels.

In adolescent females, a progressive decrease in NEFA levels was observed, being at significantly lower levels in 15-year-olds and showing no significant differences in insulin levels along the age range. A progressive increase of leptin levels was observed, with significantly lower levels in 13- year-old females.

Spearman correlation analyses between NEFA, leptin, BMI, and insulin levels by cohort and sex were performed (Table 2). Significant positive correlations (*p* < 0.001) between leptin and insulin were observed in both sexes in both age groups. Insulin correlated negatively with NEFA levels in the prepubertal group in both sexes but not in adolescents. Likewise, a significant negative correlation (*p* < 0.001) between leptin and NEFA levels was evidenced in prepubertal children but not in adolescents.

Partial correlation analyses between leptin and NEFA levels were conducted adjusting for insulin. It was observed that the negative correlation between leptin and NEFA levels remained significant in prepubertal females (−0.119, *p* = 0.023). To further explore this association, a univariate ANOVA was conducted to analyse NEFA levels across leptin tertiles, adjusting by BMI and insulin (Table 3). The analysis revealed significant differences in mean NEFA levels by leptin tertile in males (*p* = 0.013) and, particularly, in females (*p* < 0.001).

## 4. Discussion

In our study analysing plasma NEFA levels depending on age, significantly lower NEFA levels were found in 13-to-16-year-old children as compared with prepubertal children. Furthermore, a progressive decrease in NEFA levels was observed between the ages of 13 and 16 years among males and females of the adolescent cohort, while mean NEFA levels were mostly similar in children aged 6, 7, and 8 years, with only a slight decrease seen in 8-year-old females.

Data on NEFA levels in children are scarce. Published studies focus on the relationship between NEFA levels and obesity [4,5,6,7,8,22]. These studies showed age-dependent variations between NEFA levels and obesity. Valle et al. [4] and Gil-Campos et al. [7] reported lower NEFA levels in children with obesity within their prepubertal cohorts. In contrast, studies that included both prepubertal and pubertal children observed a pattern more similar to that seen in adults. For instance, Sabin et al. [6] found that children with obesity had significantly elevated fasting circulating levels of NEFA compared with normal-weight individuals, though this increase was significant only in males, not in females. Previous studies of our group have described a different association between anthropometric variables and NEFA concentrations depending on age [9,10], suggesting the age-dependent behaviour of NEFA metabolism. In this sense, studies analysing NEFA levels by age in population-based samples of children such as this one are scarce. As in our study, Allard et al. observed that plasma NEFA levels decreased with age when comparing 9-, 13-, and 16-year-old participants [23]. Our finding of lower plasma NEFA levels in adolescents as compared with prepubertal children may suggest a potential decrease in NEFA due to the increased metabolic activity and fatty acid oxidation associated with the onset of puberty, as this heightened activity sustains the physiological requirements and energy substrates necessary for growth [24].

It is worth noting that significantly higher plasma insulin levels were observed in both sexes in the adolescent cohort as compared with levels in the prepubertal group. Consequently, a down-regulation in the lipolysis process due to the anti-lipolytic activity of insulin should be present and be associated with a decrease in NEFA levels [25]. However, though a negative correlation was found between insulin and NEFA levels in prepubertal children, insulin and NEFA were not correlated in adolescents. Allard et al. reported similar results, identifying a negative correlation between NEFA and insulin levels in both 9-year-old males and females and 13-year-old males, while the correlation was not observed in 13-year-old females and in the 16-year-old group [23]. These divergences may be attributed to the decrease in insulin sensitivity occurring at puberty, as evidenced by the higher levels of plasma insulin observed in the adolescent cohort. The increase in insulin levels due to the decrease in insulin sensitivity associate to the onset of puberty has been described in several studies in children [26,27,28].

Besides this age-dependent association of NEFA with insulin, another interesting aspect in our study is the finding of a correlation of NEFA with leptin levels in prepubertal children but not in the adolescent cohort. It is well established that leptin plays a key role in maintaining energy homeostasis and regulating lipid metabolism, stimulating lipolysis and fatty acid oxidation while down-regulating lipogenesis [11,16,29]. In our study, we observed a negative correlation between NEFA and leptin levels. Given the correlation between leptin and insulin, we may think that insulin could be mediating the relationship between leptin and NEFA; however, partial correlation analysis showed that this association in prepubertal children remains significant after adjusting for insulin levels. The direct regulatory actions of leptin in the adipose tissue has been established; in vitro studies provide evidence of leptin’s direct influence on lipid metabolism in cultured isolated adipocytes, down-regulating lipogenesis and up-regulating lipolysis and fatty acid oxidation [16]. Consistent with our findings, previous research has established an association between leptin and NEFA metabolism in children, the Ulm Birth Cohort Study, which examined 8-year-old children, also demonstrated a negative association between leptin and NEFA levels [30].

The reason behind the association between NEFA and leptin levels is observed in prepubertal children but no in adolescents is struggling. This lack of correlation between NEFA and leptin in adolescents, as well as the sex differences found in our study, may be due to puberty’s impact on hormonal regulation and body composition. Puberty triggers important changes that affect fat metabolism and leptin, affecting the usual leptin-fat mass relationship. Studies have shown that insulin resistance differs by sex and Tanner stage [26], as well as described that insulin sensitivity relates to the body composition changes occurring during puberty according to sex and Tanner stage [31]. Moran A. et al. [26] demonstrated that insulin resistance differs between males and females at all Tanner stages and increases immediately at the onset of puberty, decreasing to near prepubertal levels at Tanner level T5. It seems that factors like BMI and fatness, related to leptin in younger children, do not fully explain this insulin resistance that occurs during the Tanner stages of puberty. The explanation may be related with the fact that leptin can exert its action on adipose tissue through autocrine, paracrine, or endocrine signalling pathways, acting as a programming factor during infant development with variations in the effectiveness of its action that may depend on a range of physiological conditions, underscoring its complex role in developmental processes [17]. Thus, leptin may act in an endocrine manner during prepubertal stages and then shift to an autocrine role during adolescence. Thus, during early stages, leptin activates the sympathetic nervous system through hypothalamic mechanisms, assuming pivotal roles in energy metabolism and homeostasis by releasing catecholamines [32,33], up-regulating a cascade of transcription factors that culminates in the activation of the hormone-sensitive lipase, thereby increasing lipolysis and NEFA levels [34]. Variations in plasma catecholamine levels have been reported, exhibiting disparities across sexes and pubertal stages [32,33]. The significant decrease in catecholamine levels observed with advancing puberty may be reflected in the higher plasma NEFA levels found in prepubertal children. On the other hand, as puberty advances, energy demands increase [24]. Leptin has been shown to enhance fatty acid oxidation by stimulating the activity of carnitine palmitoyltransferase-1. Although leptin promotes lipolysis within the adipocyte itself, it does not necessarily lead to an elevation of NEFA release into the bloodstream, as free fatty acids may also be used as a source of energy (ATP) or for energy storage [35].

The strengths of the study include the use of large samples from both age ranges and the fact that the subjects are representative of the population being studied within these age groups. A key limitation of our study is its cross-sectional design, which hinders our ability to establish causal relationships. Additionally, our study lacks information on physical activity levels, which can significantly influence NEFA regulation. Moreover, the study also lacks information regarding pubertal status. As pubertal changes in hormones and body composition can have a substantial impact on NEFA levels, this lack of information may affect the generalizability of our findings, in turn indicating the need to perform studies that take these changes into consideration.

## 5. Conclusions

In our study, we report lower NEFA levels in adolescents than in prepubertal children, describing a relationship of leptin with NEFA concentrations in children between the ages of 6 and 8 years, while the decrease in NEFA observed in relation to advancing age in the cohort of adolescents appears to be independent of leptin. These findings may be related to a possible different role of leptin depending on age, with an endocrine role during prepuberty that diminishes during adolescence. Further studies analysing the relationship of NEFA with leptin levels by pubertal stage would be useful to support our findings.

## Figures and Tables

**Table 1:** Anthropometric and biochemical characteristics (mean ± SD) by age and sex.

	6-to-8-Year-Olds			12-to-16-Year-Olds		
	Males (*n* = 402)	Females (*n* = 416)	*p*-Value	Males (*n* = 354)	Females (*n* = 408)	*p*-Value
Age (years)	7.1 ± 0.5	7.1 ± 0.6	0.666	14.6 ± 0.9	14.5 ± 0.8	0.341
BMI (kg/m^2^)	16.9 ± 2.3	16.9 ± 2.5	0.618	22.1 ± 4.0	21.5 ± 3.3	0.049
Z-Score BMI	−0.01 ± 1.0	0.04 ± 1.0	0.574	0.20 ± 1.1	0.08 ± 0.9	0.193
NEFA (mEq/L)	0.68 ± 0.3	0.71 ± 0.3	0.097	0.42 ± 0.2	0.44 ± 0.2	0.313
Insulin (µU/mL)	3.3 ± 2.4	3.7 ± 2.9	0.049	8.7 ± 5.8	8.4 ± 4.4	0.407
Leptin (ng/mL)	4.0 ± 4.9	6.0 ± 6.6	<0.001	6.0 ± 8.0	15.8 ± 10.0	<0.001

**Table 2:** Spearman correlation analysis of NEFA and leptin with age, BMI, and insulin in prepubertal and adolescent males and females.

**6-to-8-Year-Olds**				
	**NEFA (mmol/L)**		**Leptin (ng/mL)**	
	**>Male**	**Female**	**Male**	**Female**
BMI (kg/m^2^)	−0.076	−0.124 *	0.552 **	0.672 **
Insulin (µU/mL)	−0.231 **	−0.246 **	0.392 **	0.455 **
Leptin (ng/mL)	−0.174 **	−0.264 **	-	-
**13-to-16-Year-Olds**				
	**NEFA (mmol/L)**		**Leptin (ng/mL)**	
	**Male**	**Female**	**Male**	**Female**
BMI (kg/m^2^)	−0.057	−0.040	0.571 **	0.643 **
Insulin (µU/mL)	−0.096	−0.030	0.282 **	0.270 **
Leptin (ng/mL)	0.035	−0.062	-	-

Note: * *p* < 0.05, **p < 0.01.

**Table 3:** NEFA levels (mean ± SE) by leptin tertile adjusting by BMI and insulin in 6-to-8-year-old children by sex.

NEFA (mEq/L)				
Leptin Tertile	Males	*p*-Value	Females	*p*-Value
T1(0.07–1.53 ng/mL)	0.72 ± 0.025	0.013	0.79 ±0.03	<0.001
T2 (1.57–3.31 ng/mL)	0.62 ±0.024		0.68 ±0.03	
T3 (3.31–30.24 ng/mL)	0.64 ± 0.028		0.63 ±0.03	

## Data Availability

The original contributions presented in this study are included in the article. Further inquiries can be directed to the corresponding author.

## References

[1] Boden G. (2011). Obesity, insulin resistance and free fatty acids. Curr. Opin. Endocrinol. Diabetes Obes..

[2] Arner P., Rydén M. (2015). Fatty Acids, Obesity, and Insulin Resistance. Obes. Facts.

[3] Henderson G.C. (2021). Plasma Free Fatty Acid Concentration as a Modificable Risk Factor for Metabolic Disease. Nutrients.

[4] Valle M., Martos R., Ruz F.J., Bermudo F.M., Morales R.M., Ete R. (2002). Metabolic cardiovascular syndrome in obese prepubertal children: The role of high fasting insulin levels. Metab. Clin. Exp..

[5] Reinehr T., Kieß W., Andler W. (2005). Insulin sensitivity indices of glucose and free fatty acid metabolism in obese children and adolescents in relation to serum lipids. Metab. Clin. Exp..

[6] Sabin M.A., De Hora M., Holly J.M., Hunt L.P., Ford A.L., Williams S.R., Baker J.S., Retallick C.J., Crowne E.C., Shield J.P. (2007). Fasting Non-esterified Fatty Acid Profiles in Childhood and Their Relationship with Adiposity, Insulin Sensitivity, and Lipid Levels. Pediatrics.

[7] Gil-Campos M., Tortosa M.C.R., Aguilera C.M., Cañete R., Gil Á. (2011). Fasting and postprandial adiponectin alterations anticipate NEFA and TNF-α changes in prepubertal obese children. Nutr. Metab. Cardiovasc. Dis..

[8] Mihalik S.J., Michaliszyn S.F., Heras J.d.L., Bacha F., Lee S., Chace D.H., DeJesus V.R., Vockley J., Arslanian S.A. (2012). Metabolomic Profiling of Fatty Acid and Amino Acid Metabolism in Youth with Obesity and Type 2 Diabetes. Diabetes Care.

[9] Garcés C., Gutierrez-Guisado J., Benavente M., Cano B., Viturro E., Ortega H., de Oya M. (2005). Obesity in Spanish Schoolchildren: Relationship with Lipid Profile and Insulin Resistance. Obes. Res..

[10] Ortega L., Garcia-Anguita A., Riestra P., Ortega H., Soriano-Guillén L., Lasunción M.A., de Oya M., Garcés C. (2014). Plasma non-esterified fatty acid levels in children and their relationship with sex steroids. Steroids.

[11] Obradovic M., Sudar-Milovanovic E., Soskic S., Essack M., Arya S., Stewart A.J., Gojobori T., Isenovic E.R. (2021). Leptin and Obesity: Role and Clinical Implication. Front. Endocrinol..

[12] Harris R.B.S. (2014). Direct and indirect effects of leptin on adipocyte metabolism. Biochim. Biophys. Acta Mol. Basis Dis..

[13] Stern J.H., Rutkowski J.M., Scherer P.E. (2016). Adiponectin, Leptin, and Fatty Acids in the Maintenance of Metabolic Homeostasis through Adipose Tissue Crosstalk. Cell Metab..

[14] Casado M.E., Collado-Pérez R., Frago L.M., Barrios V. (2023). Recent Advances in the Knowledge of the Mechanisms of Leptin Physiology and Actions in Neurological and Metabolic Pathologies. Int. J. Mol. Sci..

[15] Vales-Villamarín C., Ortega-Senovilla H., de Dios O., Pérez-Nadador I., Gavela-Pérez T., Soriano-Guillén L., Garcés C. (2022). Leptin Concentration, Obesity, and Plasma Non-esterified Fatty Acid Levels in Children. Front. Pediatr..

[16] Picó C., Pomar C.A., Rodríguez A. (2021). Leptin as a key regulator of the adipose organ. Rev. Endocr. Metab. Disord..

[17] Pereira S., Cline D.L., Glavas M.M., Covey S.D., Kieffer T.J. (2020). Tissue-Specific Effects of Leptin on Glucose and Lipid Metabolism. Endocr. Rev..

[18] Kostyak J.C., Kris-Etherton P.M., Bagshaw D., DeLany J.P., Farrell P.A. (2007). Relative fat oxidation is higher in children than adults. Nutr. J..

[19] Zeng W., Pirzgalska R.M., Pereira M.M., Kubasova N., Barateiro A., Seixas E., Lu Y.-H., Kozlova A., Voss H., Martins G.G. (2015). Sympathetic neuro-adipose connections mediate Leptin-Driven lipolysis. Cell.

[20] Kraus D., Fasshauer M., Ott V., Meier B., Jost M., Klein H., Klein J. (2002). Leptin secretion and negative autocrine crosstalk with insulin in brown adipocytes. J. Endocrinol..

[21] Artalejo F.R., Garcés C., Gil Á., Lasunción M.A., José M., Moreno M., Gorgojo L., de Oya M. (1999). Four Provinces Study: Objectives and design. Rev. Esp. Cardiol..

[22] Bermúdez-Cardona J., Rodríguez C.M.V. (2016). Profile of Free Fatty Acids and Fractions of Phospholipids, Cholesterol Esters, and Triglycerides in Serum of Obese Youth with and without Metabolic Syndrome. Nutrients.

[23] Allard P., Delvin E.E., Paradis G., Hanley J.A., O’loughlin J., Lavallée C., Levy E., Lambert M. (2003). Distribution of Fasting Plasma Insulin, Free Fatty Acids, and Glucose Concentrations and of Homeostasis Model Assessment of Insulin Resistance in a Representative Sample of Quebec Children and Adolescents. Clin. Chem..

[24] Cheng H.L., Amatoury M., Steinbeck K. (2016). Energy expenditure and intake during puberty in healthy nonobese adolescents: A systematic review. Am. J. Clin. Nutr..

[25] Morigny P., Houssier M., Mouisel E., Langin D. (2016). Adipocyte lipolysis and insulin resistance. Biochimie.

[26] Moran A., Jacobs D.R., Steinberger J., Hong C.P., Prineas R., Luepker R., Sinaiko A.R. (1999). Insulin resistance during puberty: Results from clamp studies in 357 children. Diabetes.

[27] Kelsey M.M., Zeitler P.S. (2016). Insulin Resistance of Puberty. Curr. Diabetes Rep..

[28] Bloch C.A., Clemons P., Sperling M.A. (1987). Puberty decreases insulin sensitivity. J. Pediatr..

[29] Martínez-Sánchez N. (2020). There and Back Again: Leptin Actions in White Adipose Tissue. Int. J. Mol. Sci..

[30] Kirchberg F.F., Brandt S., Moß A., Peissner W., Koenig W., Rothenbacher D., Brenner H., Koletzko B., Hellmuth C., Wabitsch M. (2017). Metabolomics reveals an entanglement of fasting leptin concentrations with fatty acid oxidation and gluconeogenesis in healthy children. PLoS ONE.

[31] Travers S.H., Jeffers B.W., Bloch C.A., Hill J.O., Eckel R.H. (1995). Gender and Tanner stage differences in body composition and insulin sensitivity in early pubertal children. J. Clin. Endocrinol. Metab..

[32] Aslam S. (2020). Endocrine Events Involved in Puberty: A Revisit to Existing Knowledge. Life Sci..

[33] Weise M., Eisenhofer G., Merke D.P. (2002). Pubertal and Gender-Related Changes in the Sympathoadrenal System in Healthy Children. J. Clin. Endocr. Metab..

[34] Puente-Ruiz S.C., Jais A. (2022). Reciprocal signaling between adipose tissue depots and the central nervous system. Front. Cell Deve Biol..

[35] Diamond F., Eichler D.C. (2002). Leptin and the Adipocyte Endocrine System. Crit. Rev. Clin. Lab. Sci..

